# Research on Humanistic Quality Higher Medical Education Based on Internet of Things and Intelligent Computing

**DOI:** 10.1155/2022/8633190

**Published:** 2022-03-24

**Authors:** Rongyu Shang, Yutong Qin

**Affiliations:** Department of Medical Education, School of Basic Medicine, Army Medical University, Chongqing 400038, China

## Abstract

The importance of the humanities in promoting economic and social development is becoming increasingly clear. Combining humanities with higher medical education in order to meet the needs of medical talent training in the new situation has become a key component of higher medical education reform and development. Adult higher medical education is an integral part of higher medical education, but it has different training objectives and training objects than regular higher medical education. These technological advancements are certain to hasten the continued emergence of education cloud or industry cloud, create a good information-based environment for education informatization improvement, and pose technical challenges to resource allocation in intelligent computing environments. Humanistic quality higher medical education based on the Internet of Things and intelligent computing makes the efficient intelligent information system more open, interactive, and coordinated, allowing students and teachers to perceive a variety of teaching resources more comprehensively.

## 1. Introduction

With the development of modern science and technology, the role of humanities in promoting all-round economic and social development is becoming more and more obvious. The combination of humanities and higher medical education to meet the needs of medical talent training in the new situation has become an important content of the reform and development of higher medical education. In the era of increasingly prominent humanistic spirit, higher medical education should cultivate talents with all-round development of both humanistic quality and scientific knowledge [1]. For a long time, Chinese medical education has paid attention to the technical training of medical science and ignored the humanistic nature of medicine, resulting in the unreasonable knowledge structure of medical students and the lack of humanistic spirit. From ancient times to the present, the process of world medical education has experienced the initial integration and separation stage of humanistic and professional education and gradually moved towards a new level of integration [2, 3]. The development of medical science also poses a severe challenge to the training mode of new medical talents. The World Health Organization proposed five-star doctors as a global strategy in 1995, stating that doctors should be healthcare providers, decision makers, health educators, community leaders, and service managers in the future [4]. This necessitates not only professional knowledge but also a broad understanding of the humanities and social sciences in today's medical professionals. Adult medical college education is a vital component of higher medical education, but it differs from traditional higher medical education in terms of training objectives and objects. The key points of medical education reform in China are focusing on humanistic quality education and closing the gap between Chinese and developed countries' medical education [5].

Nowadays, with the proposal of the concept of humanistic quality higher medical education, colleges and universities across the country have carried out research on the Internet of Things and big data based on intelligent computing. These technological innovations are bound to accelerate the continuous emergence of education cloud or industry cloud and create a good information-based environment for the improvement of educational informatization. At the same time, it brings technical challenges to resource allocation in intelligent computing environment [6]. The task of the transport layer of the Internet of Things is to reliably transmit the information collected by a large number of terminals to the background central server. However, at present, the construction of the transport layer network is far from reaching the level of welcoming the explosion of the industrial scale of the Internet of Things. The massive data collected by the perception layer will flow into the transport layer network in real time, which will lead to network congestion or node failure. Therefore, how to quickly find network fault nodes is the guarantee for reliable data transmission of the Internet of Things. Intelligent computing is a powerful comprehensive computing method that combines grid computing, parallel computing, and distributed computing. Cloud computing has achieved previously unimaginable results by using server cluster [7, 8]. The campus Internet of Things construction needs to rely on powerful storage and efficient computing technology to realize higher medical education and humanistic quality education [9].

The storage and processing of massive data is the prominent feature and ability of intelligent computing [10, 11]. The intelligent computing mode of humanistic quality education system in higher medical education can ensure the security and normal operation of the data resources of the medical education platform. Students and teachers can access the medical education platform and read data at any time through the Internet [12]. Internet of Things technology, like intelligent computing technology, is network-based. The Internet of Things allows for human-computer interaction as well as human-to-human interaction. As a result, the Internet of Things is a physical Internet that can cover everything in the world [13]. The application benefits of Internet of Things technology in humanistic quality higher education and medical education are primarily manifested in that it makes the efficient intelligent information system more open, interactive, and coordinated and enables students and teachers to perceive all kinds of teaching resources more comprehensively, so as to effectively collect all kinds of required information, realize intelligent learning and teaching modes, and assist in the realization of e-learning.

## 2. Related Work

Marques et al. [14] assert that, in terms of humanistic quality, higher medical education's mastery of humanistic knowledge is not promising, as evidenced by a lack of literary knowledge, historical knowledge, philosophical knowledge, artistic knowledge, and language skills. Many domestic scholars have recognized the seriousness of the problem, conducted more research on how to inject humanistic spirit into medical science education, discussed some important theoretical problems of humanistic education in medical colleges, and made many positive explorations in methods and means [15] using the big data analysis method. According to literature [16], most higher medical education students have a strong desire and urgent need to strengthen humanistic education, improve self-cultivation and cultural taste, and perfect and realize life ideals and values. The majority of medical students are positive, but their humanistic quality needs to be improved, and humanistic education in medical schools is not given enough attention. There is a significant disparity between investment in humanistic education and the construction of teaching resources, teachers and disciplines, and a campus cultural atmosphere. According to literature [17], most medical courses are interspersed with some strong humanist courses, and the teachers who teach them are mostly former Marxism, Leninism, and ideological politics teachers. They equate humanistic education with ideological education and the cultivation of the humanistic spirit with ideological politicization, causing humanistic education to lose its vitality. Humanistic spirit cannot develop as well as science and technology in medicine. Zhu et al. [18] conducted practical research on the necessity of humanistic spirit in medical colleges from the perspective of curriculum through the method of big data analysis and came to the conclusion that medical courses must be combined with humanistic and social courses in order to cultivate talents meeting social needs. Thibaud et al.'s research [19] shows that only by integrating professional education with humanistic education can the fundamental purpose of medical respect and caring for life be restored. Mao et al. [20] pointed out that higher medical education does not have a deep understanding of the concepts of humanistic quality, humanistic quality education, humanistic spirit, etc. It is thought that the current humanistic education model has to be enhanced because it is primarily focused on a single curriculum and a single teaching technique, neither of which can inspire learning interest. The most significant humanities subjects are psychology, literature, and law, which should be made mandatory. According to the literature [21], students do not pay enough attention to humanistic quality higher education and medical education while using the big data analysis approach. 58.3% of the students are not satisfied with the humanistic quality education courses offered by the school, ignoring humanistic quality education. Due to the lack of teachers' investment, curriculum arrangement, and teaching equipment, students spend most of their time studying professional courses. Wei et al.'s research [22] shows that under the background of diversified socialist values, humanistic quality higher medical education is seriously affected by bad social atmosphere, such as money worship, hedonism, extreme individualism, and some corruption. The value orientation of medical students is somewhat deficient, and humanistic quality education is ignored, and understanding is vague. Khanna and Kaur [23] suggest that more cross courses should be added to medical education and practice in order to achieve the mutual penetration of medical humanities and medical natural sciences.

Based on the Internet of Things and intelligent computing, this paper studies the humanistic quality higher medical education, analyzes the connotation, history, function, influencing factors, and development characteristics of humanistic education in medical education, explores the training strategies of humanistic quality education for medical students under the new situation, and puts forward some suggestions and countermeasures to improve the humanistic education for medical students.

## 3. Principles and Algorithms of Internet of Things and Intelligent Computing

### 3.1. Concept and Algorithm of Internet of Things

**Figure 1:**
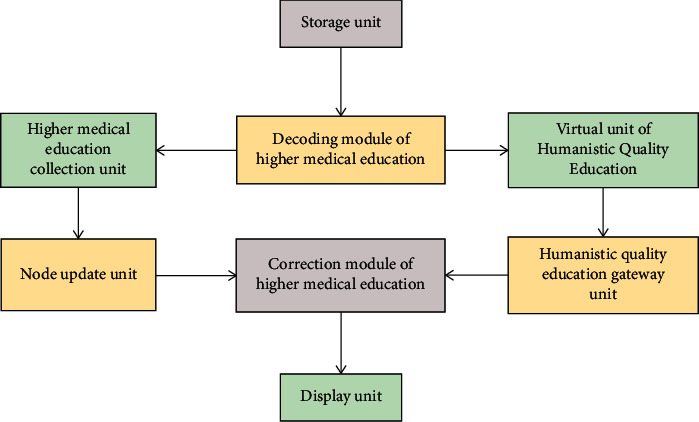
Network model of humanistic quality higher medical education.

The concept of Internet of Things was put forward by American scholars. It is a network technology that can identify, locate, track, monitor, and manage information resources. Under the Internet of Things environment, humanistic quality higher medical education is proposed, and a network model is constructed, as shown in Figure 1.

The components of the four-tier architecture of the Internet of Things are as follows.

#### 3.1.1. Perceptual Layer

The perception layer is mainly responsible for identifying objects and collecting information. The work of the perception layer needs to be completed by some terminal devices. At present, the more popular devices include two-dimensional code tags and readers, RFID tags and readers, cameras, GPS, various sensors, and so on.

#### 3.1.2. Transport Layer

The transport layer is mainly responsible for information transmission. The current mainstream technologies include communication network, wireless private network, Internet, and converged network.

#### 3.1.3. Intelligent Processing Layer

The intelligent processing layer creates the dynamic view corresponding to the physical world in the digital/virtual space mainly through processing and services such as dynamic aggregation, decomposition, and merging of sensing information.

#### 3.1.4. Application Layer

**Figure 2:**
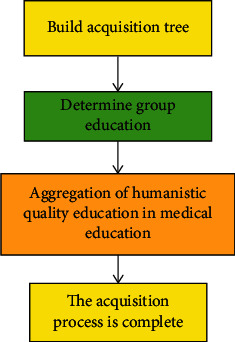
Humanistic quality education process in higher medical education.

The application layer provides rich specific services for users by using the analyzed information. However, at present, the application of the Internet of Things shows the characteristics of “narrow but small,” and it is still dominated by single and customized applications, and the sharing and use of item information is not high. The process of humanistic quality higher medical education based on the Internet of Things is shown in Figure 2.

The hierarchical clustering method is used to construct the data acquisition tree. The data sensors in each unit of the Internet of Things are regarded as a class. *N* classes are divided according to *N* units. These classes are aggregated one by one according to the distance relationship. The calculation process is shown in the following formula:

(1) $$\begin{array}{c} D_{mn}=\sum_{m-1,n-1}^{a-x,b-y}\frac{d_{ab}}{\left(x\times y\right)}. \end{array}$$

In formula (1), let the classes of hierarchical aggregation be *M*={*m*_1_, *m*_2_, *m*_3_,……, *m*_*n*_}, *N*={*n*_1_, *n*_2_, *n*_3_,……, *n*_*n*_}, the distance between *m*_*n*_ and *n*_*n*_ is dab, and the distance between classes is *D*_*mn*_. According to formula (1), a data collection tree can be constructed to provide the basis for data collection.

The distribution of data in the entire network has been understood after the data acquisition tree has been constructed using formula (1). The next step is to organize the electronic data. The grouping effect is influenced by two factors: intragroup distance AI and intergroup distance *B*_*i*_. As a result, the intragroup distance AI and intergroup distance *B*_*i*_ must be calculated in order to determine the grouping data. The following is the procedure for calculating intragroup distance AI. Calculate the total number of nodes in each group first.

(2) $$\begin{array}{c} x=\frac{\sum_{Ljex}d_{ij}}{d_{ab}\left(i,j\right)}. \end{array}$$

In formula (2), it is assumed that there are *n* data sensors in the network, which can be divided into two categories: *A* and *B*, where *I* is the degree of intragroup separation, *J* is the number of groups, *d*_*ab*_ is the distance between two groups of data, and *X* is the calculated data node. After calculating the data nodes, calculate the distance variance of two groups of data, as shown in the following formula:

(3) $$\begin{array}{c} y=\frac{\sum_{m,ney}d_{mn}}{d_{ab}\left(m,n\right)}, \end{array}$$

where *M* represents the number of nodes in the cell, *n* represents the number of data in the cell, and *Y* is the variance of data distance. Root work formulas (2) and (3) calculate the distance AI in the data group:

(4) $$\begin{array}{c} AI=\sum_{b}^{a}\frac{x}{y}, \end{array}$$

where *a*={*a*_1_, *a*_2_, *a*_3_,…, *a*_*n*_}, *b*={*b*_1_, *b*_2_, *b*_3_,…, *b*_*n*_}, *x* is the calculated data node, and *y* is the variance of data distance. The calculation process of intergroup distance BI is shown in the following formula:

(5) $$\begin{array}{c} BI=\sum_{i-1}^{m}\sum_{j-1}^{n}\left(x-y\right). \end{array}$$

The intragroup distance AI can be calculated by formula (4), and the intergroup distance *B*_*i*_ can be calculated by formula (5). When the number of groups is large, the intragroup distance AI will expand and the intergroup distance *B*_*i*_ will decrease; when the number of groups is small, the intragroup distance AI will be reduced and the intergroup distance *B*_*i*_ will be expanded. The grouping process of electronic data can be completed through the above formula.

If there is at least one path to connect ∀*v*_*i*_, *v*_*j*_ ∈ *V* in a graph *G* composed of complex networks, then *G* is called a connected graph. Because there may be many paths between nodes *v*_*i*_ and *v*_*j*_, the shortest path is called the shortest path connecting *v*_*i*_ and *v*_*j*_. Assuming that *σ*(*vi*, *vj*) is the number of shortest paths between nodes *v*_*i*_ and *v*_*j*_ and *σ*(*v*_*i*_, *v*_*j*_, *v*_*k*_) is the number of shortest paths passing through vertex *v*_*k*_ between nodes *v*_*i*_ and *v*_*k*_, then the intermediary centrality *B*(*v*_*k*_) of node VK in Figure 2 is defined as follows:

(6) $$\begin{array}{c} B\left(v_{k}\right)=\sum_{v_{i},v_{j}\in V}\frac{\sigma \left(v_{i},v_{j},v_{k}\right)}{\sigma \left(v_{i},v_{j}\right)}. \end{array}$$

As can be seen from the above definition, the more the shortest paths passing through a node, the greater its node intermediary centrality, and the shortest path in the network topology is often the path for rapid information transmission. Therefore, node intermediary centrality reflects the bearing degree of nodes on information transmission in the network.

In the figure, assuming that the degree of node *v*_*i*_ is deg (*v*_*i*_), then the sum of the degrees of all nodes in the graph is ∑_*v*_*i*_∈*V*_deg(*v*_*i*_); then, the degree centrality *D* (*v*_*i*_) of node VI is defined as

(7) $$\begin{array}{c} D\left(v_{i}\right)=\frac{\deg\left(v_{i}\right)}{\sum_{v_{i}\in V}\deg\left(v_{i}\right)}. \end{array}$$

As can be seen from the definition above, a node is a structure that connects two or more nodes together. The ratio of a node's degree to the sum of all nodes' degrees in a graph is known as degree centrality. The number of nodes directly connected to a node is referred to as its degree centrality. If a node has a high degree of centrality in a computer network, the node could be in the middle of data transmission. After a node is removed, the average shortest path is an important indicator for determining network connectivity, as it reflects the network's reliability and connectivity. The following is the definition:

(8) $$\begin{array}{c} l\equiv \left(d\left(v,w\right)\right)\equiv \frac{1}{N\left(N-1\right)}\sum_{v\in V}\sum_{w\neq v\in V}d\left(v,w\right), \end{array}$$

where *d*(*v*, *w*) is the shortest path length between two dissimilar nodes *V* and *W* in the network. In the small-world network, the value of *L* is around 6; in WWW, the value of *L* is around 17. After some nodes in the network are removed, some nodes cannot be connected, and the distance between them tends to +∞, which is a value that cannot be calculated conveniently. The concept of reverse average shortest path *L* is put forward, and its definition is as follows:

(9) $$\begin{array}{c} l\equiv \frac{1}{d\left(v,w\right)}\equiv \frac{1}{N\left(N-1\right)}\sum_{v\in V}\sum_{w\neq v\in V}\frac{1}{d\left(v,w\right)}. \end{array}$$

When two dissimilar nodes cannot communicate with each other, 1/*d*(*v*, *w*) = 0. It should be noted that *L* is not the reciprocal, and the larger the value of *L*, the better the connectivity of the network.

The size of the largest connected subgraph is the number of nodes in the largest connected subgraph in the graph, which reflects the connectivity among the nodes in the graph and is an important measure of the connectivity within the graph. Assuming that each connected subgraph set of graph *G*=(*V*, *ε*) is {*g*_1_, *g*_2_, *g*_3_, *g*_4_,…, *G*_*N*_} and there are paths between any two different nodes in these subgraphs, the relative size of the maximum connected subgraphs is defined as follows:

(10) $$\begin{array}{c} S=\frac{\text{Max}\left\{\left|V_{g1}\right|,\left|V_{g2}\right|,\left|V_{g3}\right|,...,\left|V_{gn}\right|\right\}}{\left|V\right|}, \end{array}$$

where |*V*| refers to the number of nodes in the whole computer network graph and I% *I* refers to the number of nodes contained in the connected subgraph GI.

### 3.2. Concept and Model of Intelligent Computing

**Figure 3:**
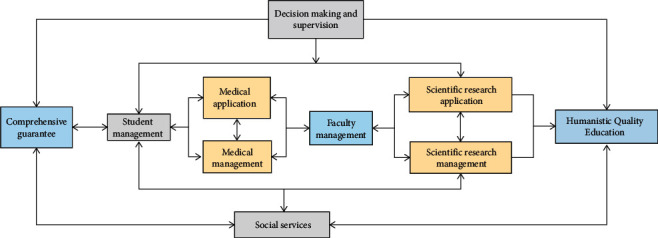
The relationship between humanistic quality education in intelligent computing higher medical education.

The development of intelligent computing technology is based on the development of network information technology. It is a computing mode, which can realize the integrated computing of a large number of data and information resources. Intelligent computing technology architecture mainly depends on three-tier architecture. The relationship among the three is closely related and interdependent, which is progressive step by step, from the bottom hardware to the application software for the end customers. Its concept is developed on the basis of SaaS (Software as a Service). In different levels of intelligent computing, people play the role of users or providers. The process of humanistic quality education in intelligent computing higher medical education involves decision making, comprehensive guarantee, financial management, student management, teaching management, teaching staff management, scientific research management, and application. Combing the business mainly solves the systematic problems of information construction, avoids more complex systematic problems caused by a single process, and provides relevant basis for the intelligent integration of various businesses. Combined with the situation of higher medical education and the development of informatization at home and abroad, Figure 3 shows the relationship between business income and humanistic quality higher education medical education.

Multiple computers can be linked together using intelligent computing technology to create a powerful technical system with massive storage and processing power. Intelligent computing's greatest advantage is that it can customize services to meet the needs of users. It will be able to meet the various network personalized service needs of school teachers, students, and teachers in this way. Intelligent computing does not alter these SAA patterns, but it does provide application providers with more options for delivering SAA products without having to configure the data center. Intelligent computing allows SaaS to be deployed without building or providing data centers, and the scale of SaaS can be scaled according to needs, just as the emergence of semiconductor OEM allows chip companies without production lines the opportunity to design and sell chips.

Intelligent computing is a revolutionary measure, which means that computing power can also be circulated as a commodity; of course, this commodity is transmitted through the Internet. The main goal of intelligent computing is to get all the services we need through network services, even including tasks such as supercomputing, with only a notebook or a mobile phone and other clients. A task space dimension can include many different resource types, and a task space can be divided into many different dimensions, and different dimensions indicate different tasks requested by users. Nowadays, intelligent computing technology has become a very popular and concerned technology in various industries, including academia. With the deepening of educational informatization construction, intelligent computing will play an increasingly important role in the construction of intelligent campus system.

## 4. Application of Humanistic Quality Higher Medical Education Based on Intelligent Computing and Internet of Things

### 4.1. Humanistic Quality Higher Medical Education in Intelligent Computing and Internet of Things

Let us compare the time required for intelligent computing and humanistic quality higher medical education. Firstly, the data blocks are divided into 5M, 60M, 120M, and 500M, respectively. On machines with the same configuration environment, serial *k*-means algorithm and intelligent computing algorithm are used to process these data blocks, respectively. The value of *k* in the experiment is 5. The experimental results are shown in Table 1.

The experimental results show that the memory occupied by *k*-means algorithm increases with the increase of the size of data file, until the memory resources are exhausted. However, parallel programs based on intelligent computing are competent, and there will be no shortage of memory. This fully proves that the intelligent computing algorithm has the ability to process large-scale data.

Intelligent computing and *k*-means algorithm are used to cluster the data of humanistic quality higher medical education, and finally different user groups are obtained. Four different data block sizes were used in this experiment, as shown in Table 2.

Medical education describes the performance of an intelligent computing algorithm as a result of a reduction in running time, which is an important metric for assessing intelligent computing performance. Medical education is the time spent on solving problems at a single node, which is the time spent on solving problems at the same node using P intelligent computing algorithms. In the following experiments, two, four, six, and eight nodes are chosen as different computing resource nodes, and their effects on the processing results are observed. The experimental results are shown in Tables 3–5.

The above table shows that increasing the number of cluster nodes reduces the processing time for the four different datasets A, B, C, and D, demonstrating that increasing the number of cluster nodes can increase the system's processing capacity. We clustered datasets of various sizes in the previous section, and the final results are similar. As a result, we only look at dataset D's clustering results. The dataset's clustering results are shown in Tables 6 and 7.

Through the Internet of Things and intelligent computing clustering, it can be seen that the online time, clicks, and network traffic generated by most mobile Internet users are very low. How to strengthen the loyalty of such users to mobile Internet services has become the primary task of network service providers.

### 4.2. Experimental Results and Analysis

**Figure 4:**
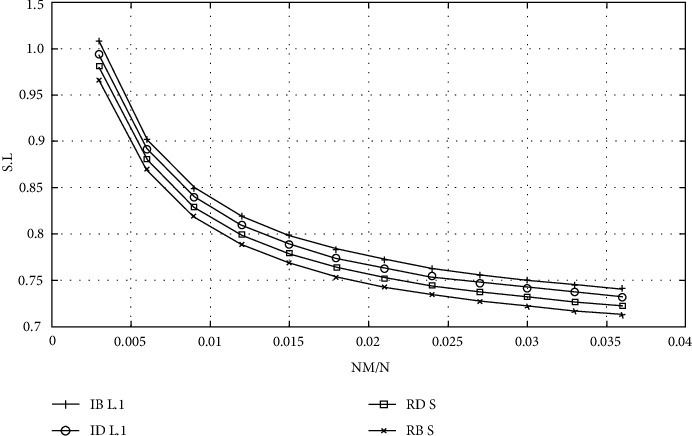
Results of S and L after simulating humanistic quality higher medical education.

**Figure 5:**
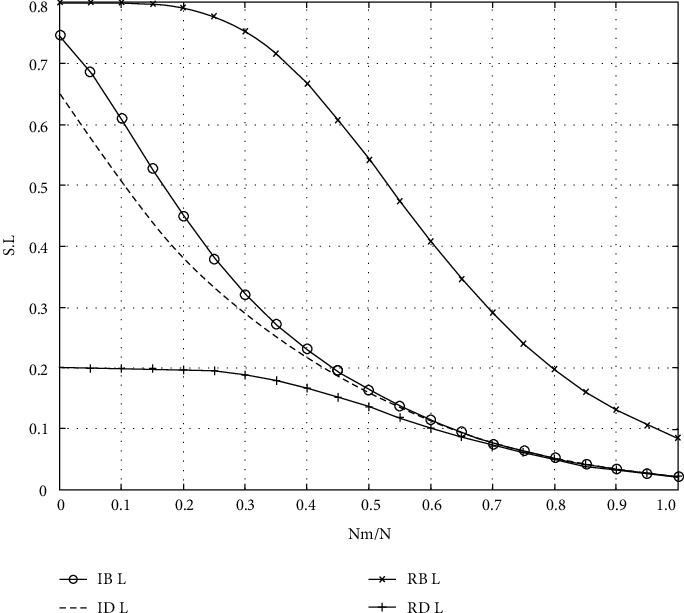
Results of S and L after simulating humanistic quality higher medical education.

Because the scale-free network accurately depicts the scale-free structure characteristics of higher medical education, it is selected as the higher medical education network simulation data set in this study. In Figure 4, the *Y* axis represents the relative values of *S* and *L* (the ratio of the values of *S* and *L* to that of the original graph is recalculated after removing the faulty nodes every time), and the data points on the curve are the results obtained by calculating the values of *S* and *L* on 210 networks after removing 51 faulty nodes in the same original BA scale-free network by four different mechanisms, as shown in Figures 4 and 5.

The following conclusions can be drawn from Figure 4. (1) For the BA scale-free network *D* with 1510 nodes, s generated by the four mechanisms is basically the same after the removal of the first 51 failed nodes. (2) When 0.76 < *l* < 0.93, the curve of Rb is the lowest after the implementation of the four mechanisms, and the influence of Rb is the largest for *L* in the whole interval.

**Figure 6:**
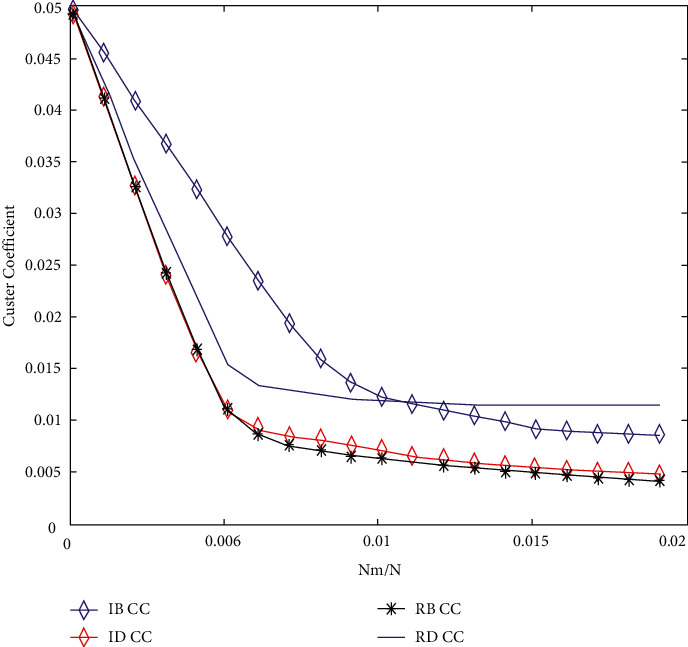
Cluster efficiency results after simulating humanistic quality higher medical education.

**Figure 7:**
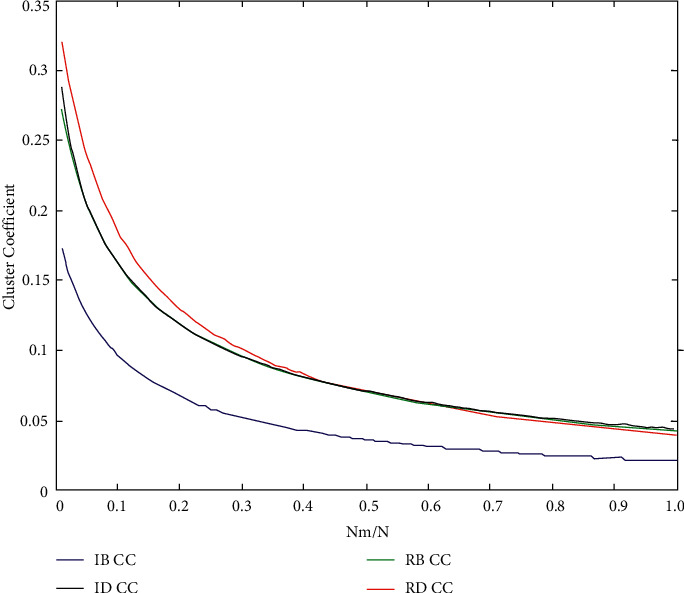
Cluster efficiency results after simulating humanistic quality higher medical education.

Figure 5 shows the graph clustering coefficient corresponding to each data point in the graph. Under normal circumstances, the higher the graph clustering coefficient, the denser the graph; otherwise, the rarer the graph. However, when the graph clustering coefficient of a curve suddenly increases from decreasing all the time, it shows that the graph suddenly produces disconnected subgraphs, which further aggravates the sparseness of the graph, as shown in Figures 6 and 7.

Figure 6 shows the following. (1) On the whole trend, IB has the greatest influence on network performance. (2) When 0.0000 < NRM/*n* < 0.0060, the effects of the four mechanisms are quite consistent. (3) When 0.0060 < NRM/*n* < 0.0300, the mechanism of IB and RB has the greatest influence, and the sudden upward trend caused by IB curve is the most obvious. The graph clustering coefficient corresponding to each data point can be obtained from Figure 7. For the whole curve, when each branch has no upward trend, IB has the greatest influence on the network performance, while for IB, it jumps the most, generates the largest number of disconnected subgraphs, and has the greatest influence.

**Figure 8:**
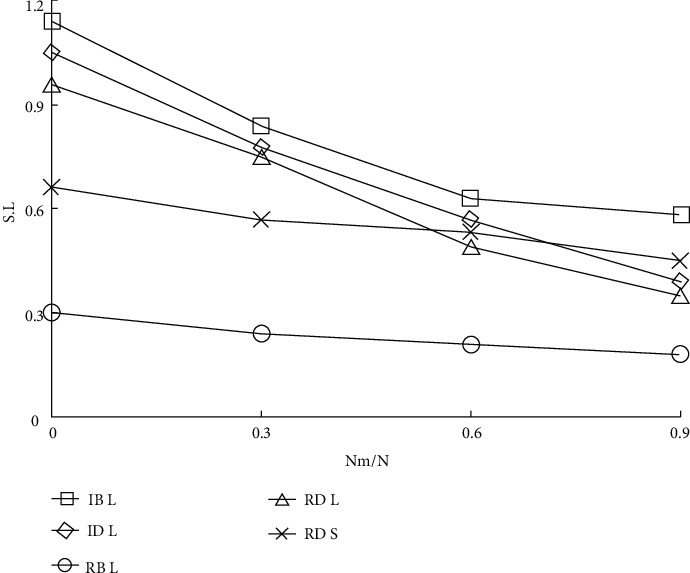
*S* and *L* results of humanistic quality higher medical education based on intelligent computing.

Three real computer network topologies are introduced to discover and analyze the impact on the actual network. The experimental results of *S* and *L* on network A are shown in Figure 8. The network model is one of the few provincial secondary trunk communication network datasets available, which, to some extent, reflects the topology characteristics of the real provincial communication network.

It can be seen from Figure 8 that for *L*, (1) when 0.1 < *l* < 1.0, Rb has the greatest impact and (2) when 0.0 < *l* < 0.1, it roughly presents the relationship of Rb > Rd > ID > IB. For the whole curve of S, Rb has the greatest impact. Because the number of nodes in the whole graph is too small, the linear relationship between S and IB is not obvious.

**Figure 9:**
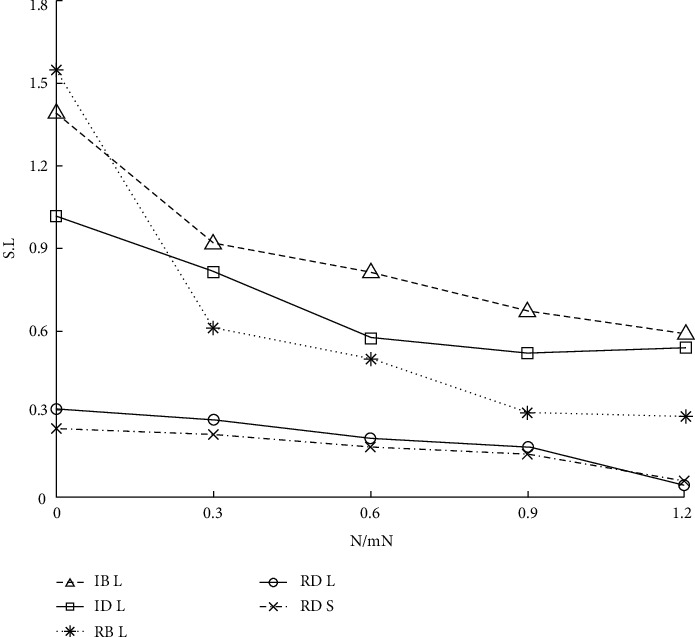
*S* and *L* results of humanistic quality higher medical education based on Internet of Things.

**Figure 10:**
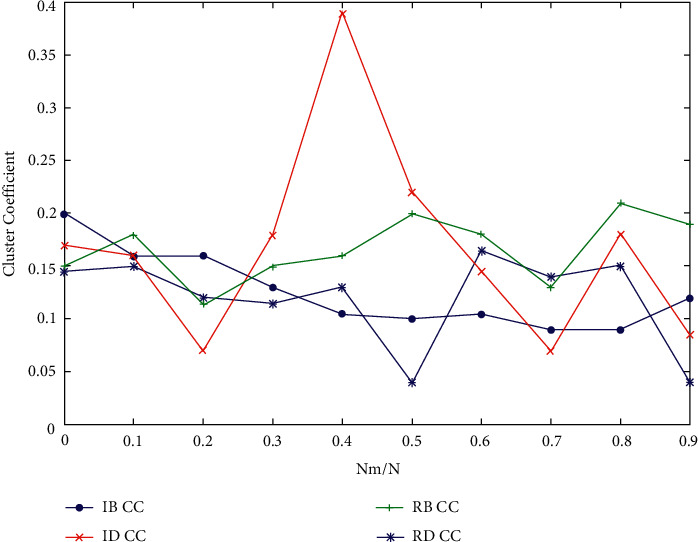
Cluster coefficient results of humanistic quality higher medical education based on Internet of Things.

Figures 9 and 10 show the experimental results of *S*, *l*, and graph clustering coefficients on network B. In Figure 9, it can be found that RB has the greatest impact on *L*, and the impact effect relationship is roughly RB > ID > Rd > IB. For the whole curve of *S*, Rb has the greatest influence, and the linear relationship between S and IB is obvious. In Figure 10, it can be found that IB has the strongest impact on network performance, with the largest number of hops, followed by RB. For the whole clustering coefficient curve, IB > RB > ID > Rd.

## 5. Conclusion

Higher education should consciously and effectively combine the cultivation of the scientific spirit of seeking truth with the cultivation of the humanistic spirit of seeking goodness, so that students understand with whom to discuss scientific truth. You know how to act when you are studying humanities. Teachers hold the key to providing high-quality humanistic education. Humanistic quality education is meaningless without high-level teaching staff. Basic medical teachers are the strongest in adult medical colleges, while humanities and social sciences teachers are the weakest. This paper examines the humanistic quality of higher medical education, examines the humanistic quality of medical education, and discusses the practicality and participation of students in humanistic education, and finally our curriculum system will incorporate humanistic spirit education into every curricular and clinical practice instruction. Strengthening teaching staff's humanistic quality construction, cultivating a group of teachers with professional skills and humanistic quality through internal training and external introduction, and intensifying cultivation can not only increase students' interest in learning but also strengthen their belief in occupation.

## Figures and Tables

**Table 1:** Comparison of processing time between intelligent computing and *k*-means algorithm.

Number of experiments	File size (M)	Number of records	t1	t2 (s)
1	5	243145	4.54s	10.368
2	35	1738310	32.76s	38.516
3	125	6953245	98.09s	144.854
4	520	290022854	Insufficient memory	638.810

**Table 2:** Experimental dataset of intelligent computing and *k*-means algorithm performance.

Dataset	Dataset size (M)	Number of data items
A	5	243145
B	35	1738310
C	125	6953245
D	520	29022856

**Table 3:** Performance experiment of humanistic quality education in medical education in Internet of Things and intelligent computing (time unit is ms).

	2	4	6	8
A	7005	4983	4065	4695
B	23258	15258	11281	9725
C	85394	52691	35647	28610
D	356241	188179	130200	98128

**Table 4:** Performance experiment of humanistic quality education in medical education in Internet of Things and intelligent computing (time unit is ms).

	2	4	6	8
A	7121	4987	4412	4745
B	23456	15245	11293	9796
C	85478	52698	35674	28674
D	356245	188487	130410	98136

**Table 5:** Performance experiment of humanistic quality education in medical education in Internet of Things and intelligent computing (time unit is ms).

	2	4	6	8
A	7584	4996	4447	4720
B	23635	15287	11345	9895
C	85745	52471	35741	28741
D	356201	188368	130689	98246

**Table 6:** Clustering result (the center point of each category).

Attribute	1	2	3	4	5
Duration	68121	15395	72036	3198	184
Clicks	34827	1434	3787	377	32
Session num	35	127	140	44	4
Uplink	2371	116	430	32	26
Downlink	825965	9997	20960	2148	167

**Table 7:** Cluster results of Internet of Things and intelligent computing (number of cases in each category).

Number of cases in each cluster	
1	4690
2	98823
3	26529
4	778333
5	28114377

## Data Availability

The data used to support the findings of this study are included within the article.
